# Localized Morbilliform Drug Eruption Following Sildenafil Exposure: A Case Report

**DOI:** 10.7759/cureus.117354

**Published:** 2026-10-02

**Authors:** Yoseph M Habte, Makida M Habte, Esimael M Abdu, Binyam M Habte

**Affiliations:** 1 Department of Medicine, Ethio Tebib Hospital, Addis Ababa, ETH; 2 Department of Medicine, Bethel Medical College, Addis Ababa, ETH; 3 Department of Surgery, Teklehaimanot General Hospital, Addis Ababa, ETH; 4 Department of Surgery, Debre Berhan University, Debre Berhan, ETH

**Keywords:** case report, cutaneous adverse drug reaction, drug-induced cutaneous reactions, morbilliform drug eruption, sildenafil

## Abstract

Sildenafil is a commonly used medication for the treatment of erectile dysfunction and is generally well-tolerated. Cutaneous adverse drug reactions associated with sildenafil are uncommon and have been described in various clinical forms. We report a case of a 34-year-old man with no significant medical history who developed an asymptomatic, localized morbilliform eruption four days after taking a single 100 mg dose of sildenafil, representing his first reported exposure. The eruption consisted of erythematous macules and papules confined to the back, without mucosal involvement, facial edema, vesiculation, bullae formation, purpura, desquamation, or systemic symptoms. Complete blood count and serum biochemical investigations were unremarkable, with no eosinophilia or evidence of hepatic, renal, or hematologic abnormalities. No other medications, supplements, recent infectious illness, or other apparent triggers were identified. Based on the clinical presentation and temporal relationship, a sildenafil-associated morbilliform drug eruption was considered. The Naranjo Adverse Drug Reaction Probability Scale yielded a score of 6, indicating a probable adverse drug reaction. The patient was treated conservatively with antihistamines, and the eruption resolved spontaneously without corticosteroid therapy or other specific interventions. This case highlights an uncommon localized morbilliform eruption following sildenafil exposure and emphasizes the importance of recognizing this potential adverse reaction.

## Introduction

Drug-induced cutaneous reactions encompass a broad spectrum of inflammatory manifestations, ranging from localized skin findings to severe reactions accompanied by systemic organ involvement. Because cutaneous manifestations may precede extracutaneous complications, the skin can provide an important early indication of an underlying systemic immune-mediated response [1,2].

Morbilliform (exanthematous) drug eruption is the most common form of cutaneous adverse drug reaction encountered in clinical practice. It is typically characterized by a symmetric eruption of erythematous macules and papules, often initially involving the trunk before extending to the extremities. The reaction generally develops within one to two weeks of starting the causative medication, although the timing may vary depending on the drug and prior exposure [1]. Earlier onset may occur in patients with previous sensitization, whereas a shorter latency following a first known exposure is less typical and may complicate clinical assessment of causality. Common culprits include antibiotics, anticonvulsants, allopurinol, antineoplastics, nonsteroidal anti-inflammatory drugs (NSAIDs), and iodinated contrast media [2]. Although uncommon, sildenafil has also been implicated in various forms of cutaneous adverse drug reactions [3,4].

We report a case of a localized morbilliform drug eruption associated with a single 100 mg dose of sildenafil in a 34-year-old man with no previous known exposure, without systemic involvement or laboratory abnormalities, which resolved with supportive treatment.

## Case presentation

A 34-year-old man with no significant medical history presented with an acute-onset skin eruption after sildenafil exposure. The patient reported taking a single 100 mg dose of sildenafil, which was obtained without a prescription prior to sexual activity. This was his first known exposure to sildenafil. He denied taking any other prescription medications, over-the-counter medications, herbal preparations, or supplements before the onset of the eruption.

Four days after sildenafil ingestion, the patient developed an asymptomatic erythematous rash localized to the back. He presented approximately one week after the onset of the eruption, during which time the lesions had evolved in appearance. The eruption remained confined to the back throughout its course. Physical examination revealed multiple erythematous macules and papules in a morbilliform pattern involving the back (Figure 1). There was no skin tenderness, vesiculation, bullae formation, purpura, facial edema, or desquamation. The oral, ocular, and genital mucosae were not involved.

**Figure 1:**
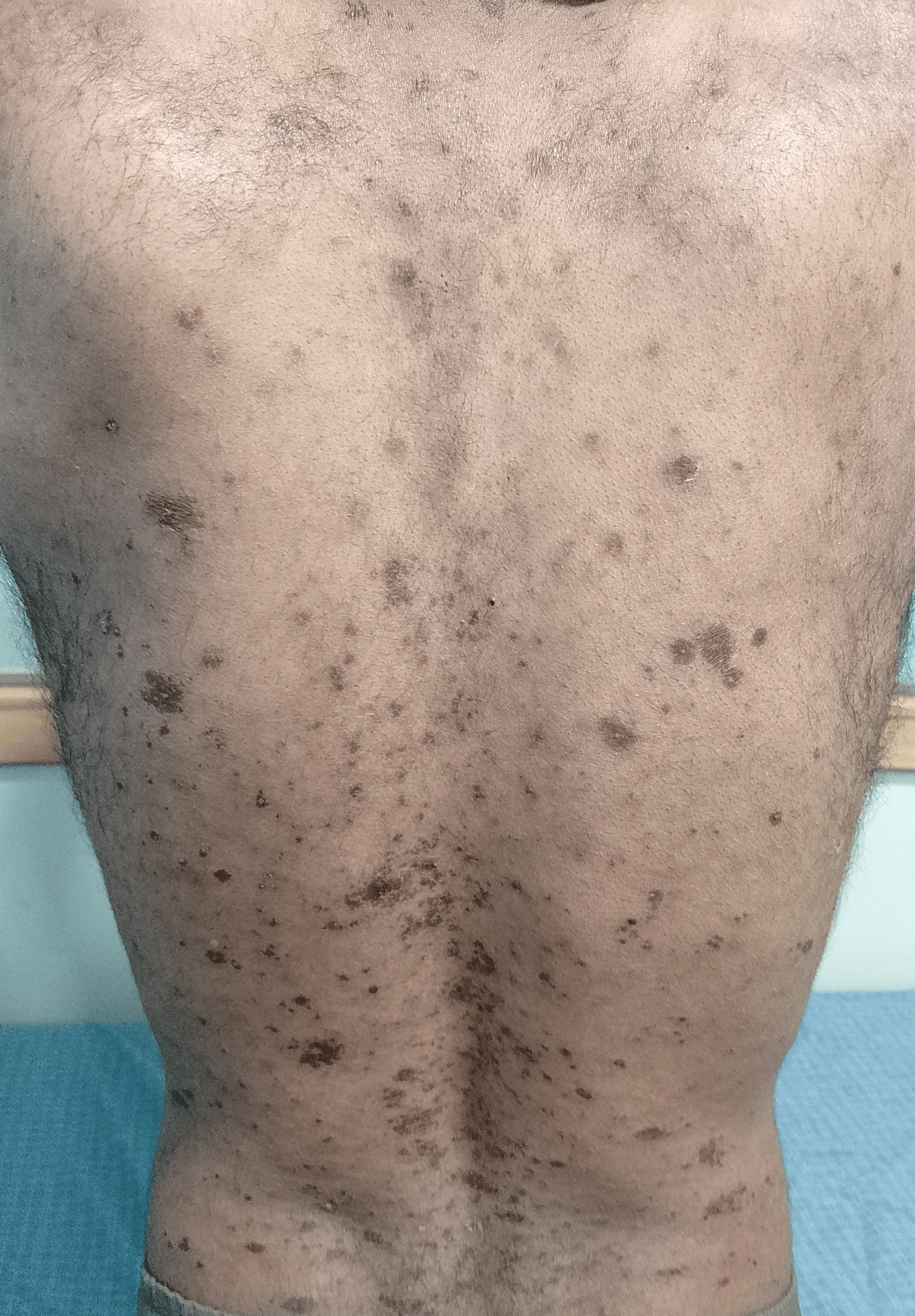
Gross photograph of the back demonstrating a localized morbilliform eruption following sildenafil exposure Grossly visible lesions in a morbilliform pattern confined to the back, photographed approximately one week after onset, showing their evolved appearance.

The patient denied systemic symptoms, including fever, malaise, arthralgia, myalgia, respiratory symptoms, gastrointestinal symptoms, or lymphadenopathy. He also reported no recent infectious illness, new dietary exposures, or additional medication use. Laboratory investigations, including complete blood count and serum biochemical tests, were within normal limits, with no eosinophilia or evidence of hepatic, renal, or hematologic abnormalities (Table 1).

**Table 1:** Laboratory investigations with corresponding results and reference values Note: Blood samples were obtained at presentation, approximately one week after rash onset.

Laboratory parameter	Result	Normal value
WBC	4.6 × 10^3^/µL	4.0-11.0 × 10^3^/µL
Hemoglobin	13.8 g/dL	13.5-17.5 g/dL
Platelet	322 × 10^3^/µL	150-450 × 10^3^/µL
Eosinophil percentage	0.1%	0%-5%
Neutrophil percentage	69.5%	45%-80%
Creatinine	0.78 mg/dL	0.6-1.0 mg/dL
Urea	14.3 mg/dL	5-18 mg/dL
Aspartate transaminase	25.2 U/L	2-50 U/L
Alanine transaminase	29.7 U/L	1-50 U/L

Based on the clinical morphology, temporal relationship with sildenafil exposure, and absence of identifiable alternative triggers, a sildenafil-associated morbilliform drug eruption was suspected. The Naranjo Adverse Drug Reaction Probability Scale yielded a score of 6, indicating a probable adverse drug reaction [5]. Points were assigned for the temporal relationship between sildenafil administration and eruption onset (+2), clinical improvement without further sildenafil exposure (+1), absence of identifiable alternative causes following clinical assessment (+2), and objective documentation of the eruption (+1). No points were assigned for rechallenge, placebo administration, drug concentration measurement, dose-response assessment, or previous exposure. Because the patient had taken only a single dose, the subsequent spontaneous improvement provides limited evidence of dechallenge, and the causality assessment should be interpreted with caution.

A skin biopsy was not performed due to the typical clinical presentation and absence of features suggestive of severe cutaneous adverse reactions requiring histopathological evaluation. The patient was managed conservatively with cetirizine 10 mg orally once daily for 5 days. The eruption had already begun to improve at the time of presentation and completely resolved approximately 21 days after onset, more than 7 days after initiation of cetirizine. Given the spontaneous improvement before treatment and the delayed complete resolution after cetirizine was discontinued, the eruption was considered to have resolved spontaneously without corticosteroid therapy or other specific interventions.

## Discussion

Sildenafil is a selective phosphodiesterase-5 (PDE5) inhibitor primarily used for the treatment of erectile dysfunction and pulmonary arterial hypertension. It has also been used off-label for conditions such as secondary Raynaud phenomenon, female sexual arousal disorder, and as adjunctive therapy for altitude-related hypoxemia [6]. Sildenafil is generally well-tolerated, with commonly reported adverse effects including headache, flushing, dyspepsia, nasal congestion, back pain, myalgia, nausea, and dizziness [6,7]. Although uncommon, sildenafil has also been implicated in cutaneous adverse drug reactions [3,4].

Morbilliform eruptions represent a characteristic cutaneous reaction pattern consisting of erythematous macules and papules that may merge into larger patches and progressively extend outward, often becoming widespread across the skin. When not accompanied by systemic manifestations, these eruptions are generally considered benign and typically resolve after withdrawal of the offending medication with appropriate supportive management [1,8,9]. Common culprit medications include antibiotics, anticonvulsants, allopurinol, antineoplastics, NSAIDs, and iodinated contrast media. The epidemiology of uncomplicated, low-risk morbilliform drug eruptions remains poorly defined, partly because there is no specific International Classification of Diseases (ICD) code that permits reliable assessment of their incidence and mortality at the population level [8].

The literature contains several case reports describing distinct cutaneous reactions associated with sildenafil, including drug reaction with eosinophilia and systemic symptoms (DRESS), erythema multiforme, and pigmented purpuric dermatosis [3,4,10]. These reactions represent different clinical manifestations from the isolated eruption observed in our patient. In contrast, our patient developed an asymptomatic, localized morbilliform eruption confined to the back, without fever, malaise, arthralgia, myalgia, respiratory or gastrointestinal symptoms, lymphadenopathy, mucosal involvement, facial edema, vesiculation, bullae, purpura, or desquamation. Laboratory investigations were also unremarkable, with no eosinophilia or evidence of hepatic, renal, or hematologic abnormalities.

Skin biopsy generally has limited utility in uncomplicated morbilliform eruptions. Histologic findings may overlap substantially among drug eruptions, graft-versus-host disease, and viral exanthems, with features such as vacuolar interface alteration, dyskeratosis, and a dermal inflammatory infiltrate that may contain eosinophils [8]. In our patient, biopsy was not performed because of the typical clinical presentation and absence of features suggestive of a severe cutaneous adverse reaction requiring histopathological evaluation.

Assessment of suspected drug causality can be strengthened through the use of standardized approaches, including the Naranjo criteria, tools specifically developed for severe cutaneous adverse reactions, such as the ALDEN score for Stevens-Johnson syndrome/toxic epidermal necrolysis, and established pharmacovigilance or drug-notoriety databases [8]. In our patient, the Naranjo Adverse Drug Reaction Probability Scale yielded a score of 6 [5], corresponding to a probable adverse drug reaction. The temporal relationship between sildenafil exposure and the onset of the eruption, absence of concomitant medications or other apparent triggers, and spontaneous resolution with supportive treatment supported the suspected association. However, the relatively short four-day latency following the patient’s first known exposure to sildenafil represents a limitation in causal attribution, as this timing is less typical of a first exposure to a morbilliform drug eruption.

The cornerstone of management of drug-induced cutaneous reactions is prompt identification and discontinuation of the suspected offending agent. In mild reactions, symptomatic treatment with topical corticosteroids and oral antihistamines is often sufficient, with symptoms typically resolving within days to weeks. Systemic therapies, including corticosteroids, immunosuppressive agents, or intravenous immunoglobulin, may be used in specialized settings according to disease severity and institutional protocols [1,9,11]. Our patient was managed conservatively with antihistamines, and the eruption resolved spontaneously without corticosteroid therapy or other specific interventions.

This case highlights an uncommon cutaneous manifestation following sildenafil exposure, characterized by a localized morbilliform eruption confined to the back without systemic or mucosal involvement following a single dose. To our knowledge, a similarly localized, uncomplicated morbilliform eruption following sildenafil exposure has not been specifically described. Recognition of this presentation is important because appropriate supportive management and avoidance of further exposure may be sufficient in patients without features suggestive of a severe cutaneous adverse reaction.

## Conclusions

Sildenafil may be associated with uncommon cutaneous adverse drug reactions, including localized morbilliform eruptions. In this case, the temporal relationship between sildenafil exposure and eruption onset, absence of alternative triggers, lack of systemic involvement, and a Naranjo score indicating probable causality supported the suspected association. The patient was managed conservatively with an antihistamine, with spontaneous resolution of the eruption. Clinicians should consider sildenafil as a potential cause of localized morbilliform eruptions, including after a first known exposure, as recognition of this potentially benign reaction may facilitate appropriate management and help distinguish it from more severe cutaneous adverse reactions, while acknowledging the limitations of causal attribution.

## References

[1] Elmehallawy GS, Alharbi RA, Kurdi SK (2026). Drug-induced cutaneous reactions: a clinically oriented review for frontline physicians. Cureus.

[2] Bettuzzi T, Sanchez-Pena P, Lebrun-Vignes B (2024). Cutaneous adverse drug reactions. Therapie.

[3] Mancano MA (2016). Sildenafil-induced erythema multiforme acute liver Injury due to febuxostat intravenous acetaminophen-induced acute hepatotoxicity acute transient myopia induced by zanamivir lidocaine-induced Hoigne syndrome. Hosp Pharm.

[4] Koçak AY, Akay BN, Heper AO (2013). Sildenafil-induced pigmented purpuric dermatosis. Cutan Ocul Toxicol.

[5] Naranjo CA, Busto U, Sellers EM (1981). A method for estimating the probability of adverse drug reactions. Clin Pharmacol Ther.

[6] Smith BP, Babos M (2023). Sildenafil. StatPearls [Internet].

[7] Chabbouh A, Rebhi F, Ben Slimene M, Jaber K, Dhaoui A (2024). Sildenafil-induced drug reaction with eosinophilia and systemic symptoms. Urol Case Rep.

[8] Yan A, Madigan L, Korman A (2025). Morbilliform eruptions: differentiating low-risk drug eruptions, severe cutaneous adverse reactions, viral eruptions, and acute graft-versus-host disease. Am J Clin Dermatol.

[9] Del Pozzo-Magaña BR, Liy-Wong C (2024). Drugs and the skin: a concise review of cutaneous adverse drug reactions. Br J Clin Pharmacol.

[10] Walterscheid B, Batchinsky M, Tarbox M (2024). Drug reaction with eosinophilia and systemic symptoms syndrome preceding toxic epidermal necrolysis secondary to sildenafil: a case report. Cureus.

[11] Sutaria A, Rawlani S, Sutaria AH (2024). Causes and management of cutaneous adverse drug reactions: a comprehensive review. Cureus.

